# Gradient-Descent-like Ghost Imaging

**DOI:** 10.3390/s21227559

**Published:** 2021-11-13

**Authors:** Wen-Kai Yu, Chen-Xi Zhu, Ya-Xin Li, Shuo-Fei Wang, Chong Cao

**Affiliations:** Key Laboratory of Advanced Optoelectronic Quantum Architecture and Measurement of Ministry of Education, School of Physics, Beijing Institute of Technology, Beijing 100081, China; 3120191480@bit.edu.cn (C.-X.Z.); 3120181429@bit.edu.cn (Y.-X.L.); 3120195769@bit.edu.cn (S.-F.W.); 3120191424@bit.edu.cn (C.C.)

**Keywords:** ghost imaging, gradient-descent, image reconstruction, iteration, image quality, denoising

## Abstract

Ghost imaging is an indirect optical imaging technique, which retrieves object information by calculating the intensity correlation between reference and bucket signals. However, in existing correlation functions, a high number of measurements is required to acquire a satisfied performance, and the increase in measurement number only leads to limited improvement in image quality. Here, inspired by the gradient descent idea that is widely used in artificial intelligence, we propose a gradient-descent-like ghost imaging method to recursively move towards the optimal solution of the preset objective function, which can efficiently reconstruct high-quality images. The feasibility of this technique has been demonstrated in both numerical simulation and optical experiments, where the image quality is greatly improved within finite steps. Since the correlation function in the iterative formula is replaceable, this technique offers more possibilities for image reconstruction of ghost imaging.

## 1. Introduction

Ghost imaging (GI) [1] is an imaging technique that produces the object image via the intensity correlation of two beams: one beam interacts with the target but is collected by a bucket detector without spatial resolution (only recording the total intensity); the other beam records the spatial distribution of the light field of the source but never interacts with the target. The detector in either beam cannot “see” the object by itself, but the object image can be recovered by calculating the intensity correlation between these two arms’ signals [2,3,4]. Later, it was found that the reference arm containing the spatially resolved array detector can be removed by using a programmable spatial light modulator (SLM) encoded with preset modulated patterns [5,6].

Over the last two decades, GI has attracted a lot of attention [7,8,9,10,11] and has been used in many fields, such as microscopic imaging [12], optical encryption [13], and cryptographic key distribution [14]. However, the signal-to-noise ratios (SNRs) of conventional GI methods are extremely low, even under a large number of measurements, and increasing the number of measurements only provides a limited improvement in image quality. To improve the image quality and imaging efficiency of the GI, many reconstruction algorithms have been proposed, such as background-removal GI (BGI) [15], high-order GI (HGI) [16,17], differential GI (DGI) [18], pseudo-inverse GI (PGI) [19], compressive GI [20,21,22], adaptive compressive GI [23], iterative GI [24,25,26], Gerchberg–Saxton-like GI [27], iterative eigenmode GI [28], joint iteration GI [29], singular value decomposition GI [30], etc. However, these methods have their limitations. The BGI finds it hard to deal with quasitransparent complex objects; the HGI relies on the increase in exponential power to improve visibility; the DGI can provide a considerable but relatively limited SNR improvement, which is mainly dependent on the object [27,31]; the PGI generally takes a long time to reconstruct large-scale object images and is sensitive to noise. The compressive GI algorithms require the sparse priors of objects and take huge matrix calculations; the adaptive compressive GI aims to reduce the sampling rate from the perspective of the object’s hierarchical structure, but has high requirements for modulated pattern design. Differing from the compressive GI method and its variants, the iterative GI, Gerchberg–Saxton-like GI, iterative eigenmode GI, joint iteration GI methods do not need to rely on the sparse priors of images, and can finish the image reconstruction task only using iterations based on statistical averages, without large-scale matrix multiplication, which undoubtedly greatly reduces computational consumption. However, these iterative variants of GI either have strict constraints or make some loose approximations of the expression of noise. In addition to these, researchers have made other attempts, such as singular value decomposition GI, but still need time-consuming calculations. Therefore, it is very necessary to construct a high-efficiency, high-quality image reconstruction method.

In this paper, inspired by the idea of classic gradient descent algorithms [32,33,34,35], we propose a new image reconstruction method, called gradient-descent-like ghost imaging (GGI). It is worth noting that the aim of the gradient descent method is to find the solution, which minimizes the objective function by updating the parameters in the opposite direction of the gradient of the objective function [32]. In addition, in view of the previous analysis, the correlation function (statistical average function) has a natural advantage as an iterative carrier, and we also know that, in GI, any form of intensity correlation functions can be regarded as a transformation function performed on the original image. Based on the above ideas, we gradually search for the optimal solution of the rewritten objective function to acquire high-quality image reconstruction. Both simulation and optical experiments will be performed to verify the performance of this proposed method against the noise without any sparse prior knowledge of the object images. The performance of this method in the recovery of complex object images will also be investigated.

## 2. Principle of Gradient-Descent-like Ghost Imaging

In this section, we will first recall the theory of gradient descent, then briefly review the common intensity correlation functions in GI and derive their matrix expression forms, and finally introduce the principle of our GGI.

### 2.1. Gradient Descent Theory

To our knowledge, gradient descent (also known as steepest descent) is a popular strategy, which is widely used in machine learning and deep learning to solve both convex and non-convex problems. Its idea is to gradually minimize an objective function, parameterized by a model’s parameters through iterations along the opposite direction of the gradient of objective function [32].

Let *J* be a function with respect to an independent vector θ, denoted as J(θ), and the gradient at $\theta_{0}$ can be written as $\nabla J(\theta_{0})$, where ∇ denotes the gradient operator. The opposite direction of the gradient direction is also called the gradient flow of the variable θ. Assume $\theta -\theta_{0}=\eta \vartheta$, where η is a positive scalar, ϑ is the unit vector of $\theta -\theta_{0}$, defined as $\vartheta =-\frac{\nabla J(\theta_{0})}{∥\nabla J(\theta_{0})∥}$. Then we can obtain $\theta =\theta_{0}+\eta \left(-\frac{\nabla J(\theta_{0})}{∥\nabla J(\theta_{0})∥}\right)=\theta_{0}-\alpha \cdot \nabla J\left(\theta_{0}\right)$, which is the core iterative expression of gradient descent. To obtain the minimum value of J(θ), the process of gradient descent can be performed via the following steps:

(1) Compute the current gradient (partial derivative) ∇J(θ) with respect to θ;
(2) Multiply the current gradient by a step size (i.e., learning rate) α, i.e., α·∇J(θ), and update the variable θ via $\theta^{\mathrm{updated}}=\theta -\alpha \cdot \nabla J\left(\theta \right)$;
(3) Repeat Steps (1–2) until the difference between the values obtained from two adjacent iterations is small enough (less than the preset termination threshold ε); at this moment, the objective function reaches its minimum;
(4) Output the current independent variable θ, which is exactly the value that minimizes the function J(θ).

For a linear measurement model, there is a hypothesis function

(1) $$h_{\theta }\left(a_{1},a_{2},\cdots ,a_{n}\right)=a_{1}\theta_{1}+a_{2}\theta_{2}+\cdots +a_{n}\theta_{n}=\Sigma_{i=1}^{n}a_{i}\theta_{i},$$

where $\theta_{i}$ (i=1,2,3,…,n) is the model parameter to be evaluated, $a_{i}$ is the coefficient or weight. To evaluate the fitting of the algorithm, a loss function can generally be used. Minimizing the loss function will help us acquire the best fitting, and the corresponding model parameters are the optimal solution. In linear regression, the loss function is usually the square of the difference between the hypothesis function and the sample output. For the convenience of derivation, we use a loss function defined as half of the mean square error (MSE):

(2) $$J\left(\theta_{1},\theta_{2},\cdots ,\theta_{n}\right)=\frac{1}{2}\mathrm{MSE}=\frac{1}{2m}\Sigma_{j=1}^{m}{\left(h_{\theta }\left(a_{1}^{\left(j\right)},a_{2}^{\left(j\right)},\cdots ,a_{n}^{\left(j\right)}\right)-y_{j}\right)}^{2},$$

where $y_{j}$ denotes the *j*th actual measured value. Then, we calculate the partial derivative of *J* with respect to $\theta_{i}$:

(3) $$\frac{\partial }{\partial \theta_{i}}J\left(\theta \right)=\frac{1}{m}\Sigma_{j=1}^{m}\left(\Sigma_{i=1}^{n}a_{i}\theta_{i}^{\left(j\right)}-y_{j}\right))a_{i}^{\left(j\right)}.$$

Thus, the iterative expression can be rewritten as

(4) $$\theta_{i}^{\mathrm{updated}}=\theta_{i}-\alpha \cdot \frac{1}{m}\Sigma_{j=1}^{m}\left(\Sigma_{i=1}^{n}a_{i}\theta_{i}^{\left(j\right)}-y_{j}\right)a_{i}^{\left(j\right)},$$

which is also called batch gradient descent [32] because it uses the gradient data of all samples when calculating the gradient.

Since each $a_{i}$ (i=1,2,3,…,n) can also be a column vector, the hypothesis function can be written as $h_{\theta }\left(A\right)=A\theta$, where $h_{\theta }\left(A\right)$ is a column vector of m×1 consisting of expected measurements, θ is a column vector of n×1 to be reconstructed, and *A* is a matrix of m×n, which can be written as $A=\left[\begin{array}{cccc} a_{11} & a_{12} & \cdots & a_{1n}\\ a_{21} & a_{22} & \cdots & a_{2n}\\ \vdots & & & \vdots \\ a_{m1} & a_{m2} & \cdots & a_{mn} \end{array}\right]$. Next, we will introduce the matrix representation of gradient descent. The foregoing loss function can be rewritten in matrix form as

(5) $$J\left(\theta \right)=\frac{1}{2m}{\left(A\theta -Y\right)}^{T}\left(A\theta -Y\right),$$

where $Y={\left[y_{1},y_{2},\cdots ,y_{m}\right]}^{T}$ is a column vector of m×1, which consists of actual measurements (sample outputs), and *T* denotes the transposition operator. Then, the partial derivative of J(θ) with respect to θ can be computed via

(6) $$\frac{\partial }{\partial \theta }J\left(\theta \right)=\frac{\partial }{\partial \theta }\frac{1}{2m}\left(\theta^{T}A^{T}A\theta -\theta^{T}A^{T}Y-Y^{T}A\theta +Y^{T}Y\right)=\frac{1}{m}A^{T}\left(A\theta -Y\right).$$

Thus, the updated expression of θ can be rewritten as

(7) $$\theta^{\mathrm{updated}}=\theta -\alpha \cdot \frac{1}{m}A^{T}\left(A\theta -Y\right).$$

**Figure 1:**
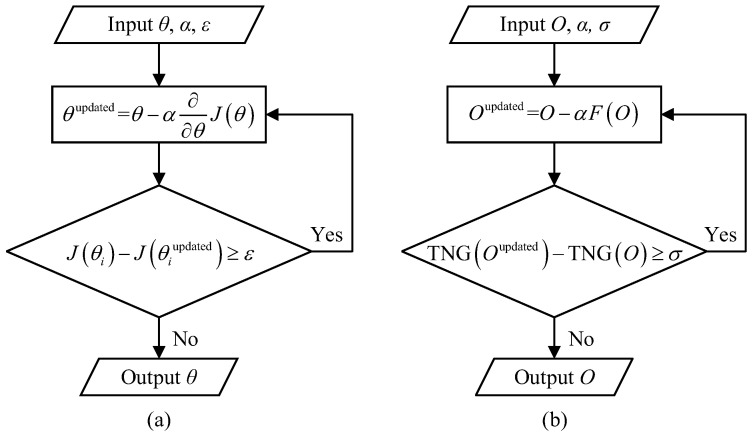
Algorithm flow chart. (**a**) Flow chart of the gradient descent idea. (**b**) Flow chart of our gradient-descent-like ghost imaging algorithm.

Figure 1a shows the schematic diagram of gradient descent. The minimum value of J(θ) can be obtained by iterating along the direction of gradient descent. First, it is worth mentioning that the value α represents the length of each step in the gradient direction during iterations. If the value α is too large, we cannot guarantee that the gradient will be decreased in each iteration, nor can we guarantee the convergence. If the value α is too small, it will lead to a painfully slow convergence and long calculation time, but the update value can achieve almost an optimal solution to the objective function, as it is unlikely that the stepping will miss any useful gradient position. Thus, this value α determines the convergence speed of the iterations and whether the iterations can reach the optimal solution. Second, if the function J(θ) is convex, the iterative result starting from one initial value will be the optimal solution by a large probability; if J(θ) is non-convex, it is necessary to perform the gradient descent strategy multiple times, each with different initial values, to get rid of the local optimal solution, and then the solution with the smallest functional value should be selected from these iterative results. Generally, different initial values may lead to different minimum values of the objective function. Therefore, we should carefully choose the initial value of iterations, as well as the iteration step size.

### 2.2. Intensity Correlation Functions in GI

In GI, the object $\hat{O}\left(x,y\right)$ is illuminated by several modulated patterns $I_{R}\left(x,y\right)=\left\{I_{R}^{1}\left(x,y\right),I_{R}^{2}\left(x,y\right),\cdots ,I_{R}^{j}\left(x,y\right),\cdots ,I_{R}^{m}\left(x,y\right)\right\}$, where the superscript j=1,2,3,⋯,m denotes the *j*th modulation, *x* and *y* stand for the spatial coordinates on *x*-axis and *y*-axis. The total intensity collected by the bucket detector can be written as $S_{B}=\int I_{R}\left(x,y\right)\hat{O}\left(x,y\right)dxdy$. The object image can be retrieved by calculating the intensity correlation between the patterns $\left\{I_{R}^{1}\left(x,y\right),I_{R}^{2}\left(x,y\right),\cdots ,I_{R}^{m}\left(x,y\right)\right\}$ and bucket values $S_{B}=\left\{S_{B}^{1},S_{B}^{2},\cdots ,S_{B}^{m}\right\}$. In the following, we will briefly introduce BGI, HGI, DGI, logarithmic GI (LGI), trigonometric ghost imaging (TGI) [36].

First of all, the most classic second-order correlation in GI can be written as

(8) $$G^{\left(2\right)}\left(x,y\right)=\langle S_{B}I_{R}\left(x,y\right)\rangle ,$$

where $\langle u\rangle =\frac{1}{m}\sum_{j=1}^{m}u^{j}$ stands for the ensemble average of the signal *u*. It is worth mentioning that this classic second-order correlation function is a prototype algorithm of GI. Obviously, this function is too simple to obtain a satisfactory image quality, even with a large number of measurements, and the reconstructed image contains non-negligible background noise (which can be treated as the direct component). Therefore, this formula has basically withdrawn from history, but its derivatives have gradually become the mainstream algorithms.

For example, based on the above formula, by making $S_{B}$ and $I_{R}\left(x,y\right)$ separately subtract the average terms of $S_{B}$ and $I_{R}\left(x,y\right)$, we can acquire the functional form of the BGI [15]:

(9) $$\begin{array}{cc} G_{\mathrm{BGI}}\left(x,y\right) & =\langle \left(S_{B}-\langle S_{B}\rangle \right)\left(I_{R}\left(x,y\right)-\langle I_{R}\left(x,y\right)\rangle \right)\rangle \\ \; & =\langle S_{B}I_{R}\left(x,y\right)\rangle -\langle S_{B}\rangle \langle I_{R}\left(x,y\right)\rangle . \end{array}$$

Thus, this formula uses $\langle S_{B}\rangle \langle I_{R}\left(x,y\right)\rangle$ to describe the background noise. By subtracting this product term from $\langle S_{B}I_{R}\left(x,y\right)\rangle$, the BGI can generate a good-quality image for simple objects with high transmittance, but fails to work in the case of unstable light sources (e.g., with temperature drift) or quasitransparent complex objects.

By calculating high-order correlation [16], we can obtain

(10) $$G_{\mathrm{HGI}}\left(x,y\right)=\langle {\left(S_{B}\right)}^{p}{\left(I_{R}\left(x,y\right)\right)}^{q}\rangle ,$$

where *p* and *q* are the power indices of the bucket and reference signals, respectively. The HGI can improve the visibility and contrast by selecting appropriate *p* and *q* values, but it cannot effectively remove background noise.

By replacing $\langle S_{B}\rangle$ in BGI with a new differential term $\frac{\langle S_{B}\rangle }{\langle S_{R}\rangle }S_{R}$ ($S_{R}=\int I_{R}\left(x,y\right)dxdy$), which reflects the relative fluctuations in the bucket values, we will obtain the functional form of the DGI [18]:

(11) $$\begin{array}{cc} G_{\mathrm{DGI}}\left(x,y\right) & =\langle \left(S_{B}-\frac{\langle S_{B}\rangle }{\langle S_{R}\rangle }S_{R}\right)\left(I_{R}\left(x,y\right)-\langle I_{R}\left(x,y\right)\rangle \right)\rangle \\ \; & =\langle S_{B}I_{R}\left(x,y\right)\rangle -\frac{\langle S_{B}\rangle }{\langle S_{R}\rangle }\langle S_{R}I_{R}\left(x,y\right)\rangle . \end{array}$$

Compared with the BGI, the term $\frac{\langle S_{B}\rangle }{\langle S_{R}\rangle }\langle S_{R}I_{R}\left(x,y\right)\rangle$ in the DGI can better describe the background noise. Therefore, the DGI can more accurately remove background noise, and significantly improve the image quality, even in harsh or noisy measurement environments.

The expression of the LGI is defined [36] as follows:

(12) $$G_{\mathrm{LGI}}\left(x,y\right)=\langle \log_{C}\frac{S_{B}}{\langle S_{B}\rangle }I_{R}\left(x,y\right)\rangle ,$$

where *C* is the base of the logarithm function. According to the logarithmic operational rule, the above formula can also be rewritten as $G_{\mathrm{LGI}}\left(x,y\right)=\langle \log_{C}S_{B}I_{R}\left(x,y\right)\rangle -\langle \log_{C}\langle S_{B}\rangle \rangle$; thus, some part of background noise is removed.

The formula of the TGI [36] can be written as

(13) $$G_{\mathrm{TGI}}\left(x,y\right)=\langle \left[\sin\left(d+S_{B}^{\prime }-\frac{1}{2}\right)\pi \right]I_{R}\left(x,y\right)\rangle ,$$

where $S_{B}^{\prime }=\left(S_{B}-S_{B}^{\min}\right)/\left(S_{B}^{\max}-S_{B}^{\min}\right)$, $S_{B}^{\max}$ and $S_{B}^{\min}$ are the maximum and minimum of $S_{B}$, respectively. Here, *d* takes even integers to generate positive images [36]. Since the TGI only performs a triangular transformation on $S_{B}^{\prime }$ on the basis of the classic second-order correlation function, the reconstruction quality obtained via TGI is not very different from the recovered result of the classic second-order correlation function.

We find that Equations (8)–(13) can be divided into two categories: functions without subtractive background terms and functions with subtractive background terms. The first category, containing the classic second-order correlation function, HGI and TGI, mainly focuses on enhancing the image visibility by optimizing bucket values, modulated patterns or both; the second category, including the BGI, DGI and LGI, tries to characterize the background noise and subtracts it from the reconstructed image of the classic second-order correlation function to improve the image quality. Among them, the DGI is recognized as the best statistical correlation algorithm due to its excellent and stable imaging performance.

To simplify the derivation, we will also derive the matrix forms of these functions. Each modulated pattern can be reshaped into a row vector of 1×n, *m* row vectors reshaped from *m* patterns will form a measurement matrix *A* of m×n. In the same way, the object image $\hat{O}$ can be flattened into a column vector *O* of n×1. Thus, the ideal bucket values can be written as AO, and the actual bucket values will be denoted by $V={\left[S_{B}^{1},S_{B}^{2},\cdots ,S_{B}^{m}\right]}^{T}$. In the ideal measurement environment, V=AO. Then, we will have $\langle S_{B}\rangle =\frac{1}{m}V^{T}E_{m\times 1}=\frac{1}{m}E_{1\times m}V$, $\langle I_{A}\rangle =\frac{1}{m}A^{T}E_{m\times 1}$, $S_{A}=AE_{n\times 1}$, $\langle S_{A}\rangle =\frac{1}{m}E_{1\times m}AE_{n\times 1}$, where *E* denotes a matrix consisting of all ones and its subscripts stand for the dimensions of this matrix. In the following, we can rewrite the above intensity correlation functions as

(14) $$F_{G^{\left(2\right)}}\left(O\right)=\frac{1}{m}A^{T}AO,$$

(15) $$F_{G_{\mathrm{BGI}}}\left(O\right)=\frac{1}{m}A^{T}AO-\frac{1}{m^{2}}E_{1\times m}AOA^{T}E_{m\times 1},$$

(16) $$F_{G_{\mathrm{HGI}}}\left(O\right)=\frac{1}{m}{\left(A^{T}\right)}^{.q}{\left(AO\right)}^{.p},$$

(17) $$\begin{array}{cc} F_{G_{\mathrm{DGI}}}\left(O\right) & =\frac{1}{m}A^{T}AO-\frac{\frac{1}{m}E_{1\times m}V}{\frac{1}{m}E_{1\times m}AE_{n\times 1}}\cdot \frac{1}{m}A^{T}AE_{n\times 1}\\ \; & =\frac{1}{m}A^{T}A\left(O-\frac{E_{1\times m}AOE_{n\times 1}}{E_{1\times m}AE_{n\times 1}}\right)\\ \; & =\frac{1}{m}A^{T}A\left(O-\tilde{O}\right)\\ \; & =\frac{1}{m}A^{T}\left(AO-A\tilde{O}\right)\\ \; & =\frac{1}{m}A^{T}\left(AO-\tilde{V}\right), \end{array}$$

(18) $$F_{G_{\mathrm{LGI}}}\left(O\right)=\frac{1}{m}A^{T}\left(\log_{C}\frac{AO}{\frac{1}{m}E_{1\times m}AO}\right),$$

(19) $$F_{G_{\mathrm{TGI}}}\left(O\right)=\frac{1}{m}A^{T}\left(\sin\left(d+V^{\prime }-\frac{1}{2}\right)\pi \right),$$

where .q and .p denote the powers that are performed on each element of the matrix, $\tilde{O}=\frac{E_{1\times m}AOE_{n\times 1}}{E_{1\times m}AE_{n\times 1}}$ characterizes the average light field, $\tilde{V}=A\tilde{O}$, and $V^{\prime }=\left(V-V_{\min}\right)/\left(V_{\max}-V_{\min}\right)$. Although the above derivations are carried out under noise-free conditions, the actual recorded bucket values are generally accompanied by the scaling of light intensities as well as measurement noise *e*, i.e., V=κAO+e, where κ denotes the proportional coefficient determined by the light attenuation, collection efficiency, photoelectric conversion efficiency, etc. However, this does not affect the above derivations because, for $V=\kappa AO+e=A\left(\kappa O+e^{\prime }\right)=AO^{\prime }$, where $O^{\prime }=\kappa O+e^{\prime }$, we only need to replace original *O* with $O^{\prime }$.

### 2.3. Gradient-Descent-like Ghost Imaging

Next, let us first review the composition of the original image *O*. Generally, the image *O* can be considered to be composed of the object part $O_{o}$ and background part $O_{b}$ (a direct component), i.e., $O=O_{o}+O_{b}$ (the values in both $O_{o}$ and $O_{b}$ are greater than 0); thus, we will have $AO=AO_{o}+AO_{b}$. According to the previous classification, it is easy to notice that $F_{G_{\mathrm{BGI}}}\left(O\right)$, $F_{G_{\mathrm{DGI}}}\left(O\right)$, $F_{G_{\mathrm{LGI}}}\left(O\right)$ are in forms of $\frac{1}{m}A^{T}\left(AO-\tilde{\Omega }\right)$. In terms of GI principle, we expect to make the element values of this subtractive term $\tilde{\Omega }$ as close to those of $AO_{b}$ as possible. The closer these two sets of values are, the higher the reconstruction quality of the intensity correlation function is. We find that the form of $\frac{1}{m}A^{T}\left(AO-\tilde{\Omega }\right)$ looks very similar to the gradient form $\frac{\partial }{\partial \theta }J\left(\theta \right)=\frac{1}{m}A^{T}\left(A\theta -Y\right)$ of the traditional gradient descent algorithm. If we regard the above three intensity correlation functions as the image gradients $\frac{\partial }{\partial O}J\left(O\right)$, then their primitive functions J(O) can be written as the transform forms of $J\left(O\right)=\frac{1}{2m}{\left(AO-\tilde{\Omega }\right)}^{T}\left(AO-\tilde{\Omega }\right)$. $F_{G_{\mathrm{DGI}}}\left(O\right)$ happens to be the gradient of the loss function $J\left(O\right)=\frac{1}{2m}{\left(AO-\tilde{V}\right)}^{T}\left(AO-\tilde{V}\right)$ without any transformation. Different from the loss function (e.g., $J\left(\theta \right)=\frac{1}{2m}{\left(A\theta -Y\right)}^{T}\left(A\theta -Y\right)$) in traditional gradient descent algorithm, here, J(O) is to make $\tilde{\Omega }$ as close to a direct component $AO_{b}$ (rather than AO) as possible so that J(O) will reach its minimum near the optimal solution (while in classic gradient descent algorithm, J(θ) is to make *Y* as close to Aθ as possible so that the residual error can be minimized; thus, both J(θ) and its gradient will vanish to 0 near the optimal solution). However, it does not affect the correlation functions being regarded here as the gradients, because, no matter whether J(θ) or J(O) is used, the ultimate goal is to minimize the objective function by reducing the gradient value. Following this idea, $A^{T}\tilde{\Omega }\to A^{T}AO_{b}$ tries to characterize the background noise, so $A^{T}AO-A^{T}\tilde{\Omega }$ intends to extract the object part, and the values of $\frac{1}{m}A^{T}\left(AO-\tilde{\Omega }\right)$ are expected to be continuously reduced to make the reconstructed object part closer to the optimal solution. $F_{G_{\mathrm{HGI}}}\left(O\right)$ and $F_{G_{\mathrm{TGI}}}\left(O\right)$ can be expressed as the transform forms of $F_{G^{\left(2\right)}}\left(O\right)$ ($\frac{1}{m}A^{T}AO$), namely, to make the subtractive term $\tilde{\Omega }$ in $\frac{1}{m}A^{T}\left(AO-\tilde{\Omega }\right)$ equal to zero, but with relatively poor imaging performance. Therefore, most of the intensity correlation functions (especially the ones with subtractive background terms) can be regarded as the gradients that are expected to be minimized.

According to the above theoretical analysis and proof, we propose a GGI method. It has been proven that, no matter what correlation function we use to recover the object image, the probability of recovered pixel values located in the pixel region of the same original gray value of the object follows a Gaussian distribution, whose average value has a linear relationship with this given original gray value [31,37,38]. For this reason, all intensity correlation algorithms can be regarded as the functions *F* of the object image $\hat{O}$, denoted as $F(\hat{O})$. Here, we let F(O) (with respect to the current estimate image *O*) represent the above intensity correlation functions. F(O) can be regarded as the gradient of J(O), i.e., the partial derivative $\frac{\partial }{\partial O}J\left(O\right)$. Since the original object image to be recovered is generally unknown in advance, J(O) will fail to work because its change cannot be observed intuitively. Fortunately, there are many equivalent loss functions available as alternatives to assert the convergence of the iterations in a direct way, such as $∥O_{k}-O_{k-1}{∥}_{0}$, $∥O_{k}-O_{k-1}{∥}_{1}$, $∥O_{k}-O_{k-1}{∥}_{2}$, $∥O_{k}-O_{k-1}{∥}_{\mathrm{TV}}$ and no-reference image quality assessment, where TV is short for total variation. Here, we choose to use Tenengrad (TNG) function [39,40] (a similar method to the TV norm) as the algorithm’s termination criterion, which calculates the sum of the squares of all pixels’ gradient values in a current estimate that are greater than a certain threshold, and is defined [39] as

(20) $$\mathrm{TNG}=\sum_{x}\sum_{y}{\left[G\left(x,y\right)\right]}^{2},\;\;\;\mathrm{for}\;\;G\left(x,y\right)>\gamma ,$$

where γ is the preset threshold. In this function, G(x,y) is the gradient, which can be written as

(21) $$G\left(x,y\right)=\sqrt{\left(f_{x}^{2}+f_{y}^{2}\right)},$$

where $f_{x}$ and $f_{y}$ are the gradients along *x* and *y* axes, respectively, which can be obtained by the well-known Sobel (discrete differentiation) operator. Generally, the sharper the edge of the natural image, the higher the gradient value (before computing the value of TNG, the column vector *O* needs to be reshaped into a matrix form). In its implementation, we only need to compare the difference between the values of the TNG function obtained by two adjacent iterations to determine whether the algorithm has converged.

Figure 1b gives the algorithm flow chart of the proposed GGI, and its algorithm steps can be described as follows:

(1) Compute the current F(O) with respect to the current estimate *O*;
(2) Let *O* minus α times the current estimate obtained in Step (1) to produce an updated estimate $O^{\mathrm{updated}}$:
  (22) $$O^{\mathrm{updated}}=O-\alpha F\left(O\right);$$
(3) Judge whether the difference between $\mathrm{TNG}\left(O^{\mathrm{updated}}\right)$ and TNG(O) is greater than a predefined threshold σ (which is set to $10^{-10}$ in this work): if not, the algorithm will be terminated, the current estimate *O* is the optimal solution, which will then be reshaped to an image matrix *U*; if so, replace *O* with $O^{\mathrm{updated}}$ and repeat Steps (1–2);
(4) Output the reconstructed image *U*.

It should be noted that the main iterative formula as given in Equation (22) is only related to the current estimate *O* (a possible solution to the recovered ghost image), predefined threshold α and newly computed function F(O), while intensity correlation functions depend on the current bucket values and modulated patterns. This means that the function F(O) in Equation (22) can be replaced with intensity correlation functions, with the inputs serving as the modulated patterns and the new bucket values calculated with the current estimate *O*. In other words, in each iteration, the modulated patterns remain unchanged, while the bucket values are updated according to the current estimate *O*. Therefore, the bucket values used in the first iteration are obtained from actual measurements, while the ones used in the subsequent iterations are numerically calculated. In addition, since the intensity correlation function F(O), loss function J(O) and its equivalent TNG function are all convex, by following the direction of the slope of the convex surface created by the loss function downhill, the iterations that start from some initial values tend to reach a valley, and the (local) optimal solution will be acquired after multiple iterations by a very large probability according to the theory of gradient descent. The iterative formulas that use intensity correlation functions with subtractive background terms will definitely achieve better convergence than the cases using intensity correlation functions without subtractive background terms, because the gradient functions in the former cases will tend towards the minimum near the optimal solution and are more conducive to the convergence of iterations.

Next, for the sake of simplification, we abbreviate the gradient-descent-like BGI, gradient-descent-like HGI, gradient-descent-like DGI, gradient-descent-like LGI and gradient-descent-like TGI as the GBGI, GHGI, GDGI, GLGI, GTGI, respectively.

## 3. Simulation Results and Analysis

To demonstrate the performance of the proposed method, we used random binary patterns for numerical simulations. Here, we set p=10 and q=1 for the HGI function, as a large *p* will increase the image visibility and a small *q* will dramatically suppress the noise. For LGI, the base of the logarithmic function is set to C=10, while, in TGI, we set d=0.

**Figure 2:**
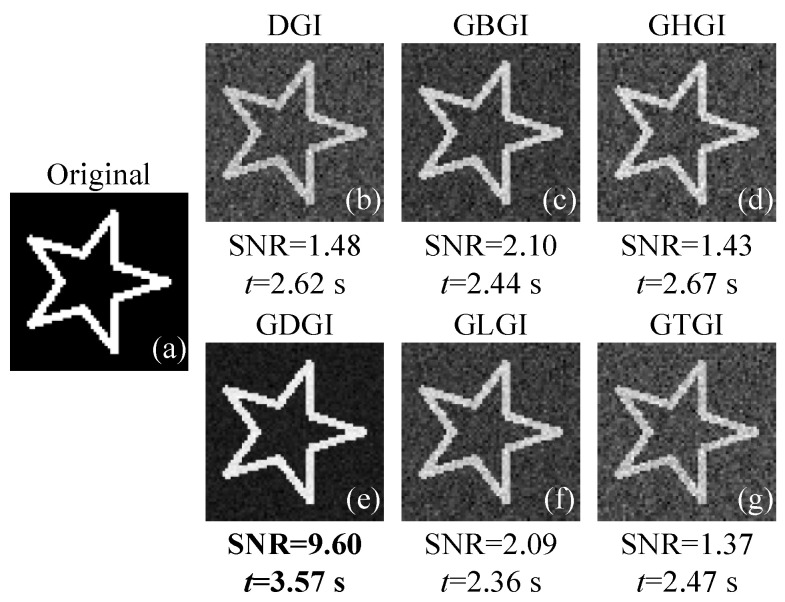
Simulation results of the binary image “01” using different ghost imaging (GI) algorithms with m=20,480 measurements. (**a**) is the original image “01”, (**b**–**g**) are the images recovered by the differential GI (DGI), gradient-descent-like background-removal GI (GBGI), gradient-descent-like high-order GI (GHGI), gradient-descent-like DGI (GDGI), gradient-descent-like logarithmic GI (GLGI) and gradient-descent-like trigonometric GI (GTGI), respectively, with their signal-to-noise ratios (SNRs) and running time *t* marked below the figures.

Figure 2a shows the binary image labelled “01” consisting of 64×64 pixels. The DGI, GBGI, GHGI, GDGI, GLGI and GTGI are all computed with 20,480 measurements and the step size α is set at 0.1 (which will be explained below). It should be noted that all these reconstruction algorithms are based on statistical intensity correlation, which means that oversampling is generally required in classic GI functions, but the large-scale matrix multiplication and prior knowledge of image sparsity are no longer needed, so the computational complexity is very low. Although oversampling is not as efficient as a simple scanning, GI, as an indirect computational imaging technique, only needs to record the total light intensity, which can effectively avoid the average distribution of luminous flux across dimensions, as in point scanning. Especially under ultra-low-light illumination, this advantage will become more prominent. In addition, GI has a better anti-turbulence and a better imaging sensitivity than scanning. Since the focus of this manuscript is more on how to improve the imaging quality of the intensity correlation functions through only a few iterations, the number of measurements used here (20,480) is just an example to illustrate the performance improvement, and the subsampling cases will be discussed later. The reconstruction results of the above algorithms are given in Figure 2b–g, with their SNRs and running time *t* marked right below the figures. The algorithms are running under the operating system Windows 10, with the memory of 16.0 GB and the central processor AMD Ryzen 7 4800H of 2.90 GHz integrated with Radeon Graphics. It can clearly be seen from the simulation results that the SNRs of the GBGI, GDGI, and GLGI are higher than those of the GHGI and GTGI (consistent with our previous theoretical analysis), which means that they gradually approach the position of the optimal solution after a few iterations. We note that the SNR of the GHGI is slightly lower than that of DGI, because the high-order operation of the bucket values amplifies the noise while enhancing the signal. The GTGI also fails to acquire the performance gain, mainly due to the absence of subtractive background term. It is worth noting that, among these, the SNR of the GDGI can reach 9.60, which is several times that of the DGI. The main reason the GDGI can outperform other variants is that the DGI function expression it uses happens to be $\frac{1}{m}A^{T}\left(AO-\tilde{V}\right)$, whose primitive function is exactly the loss function $J\left(O\right)=\frac{1}{2m}{\left(AO-\tilde{V}\right)}^{T}\left(AO-\tilde{V}\right)$ without any transformation. Besides this, the calculation time of the GDGI is 3.57 s, with only a small increase in computing time compared to the DGI, which is acceptable in most cases. Therefore, the GDGI can achieve a much better reconstruction performance without much increase in computing time compared with the DGI.

**Figure 3:**
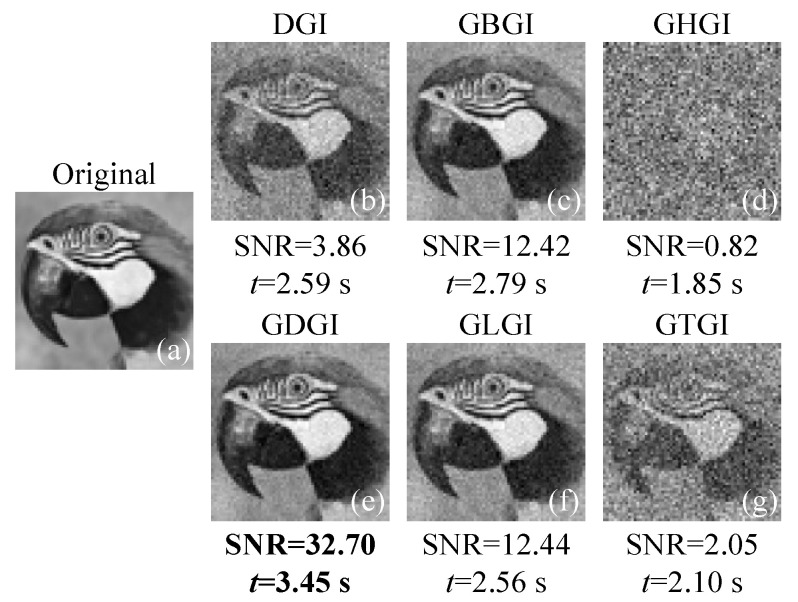
Simulation results of the grayscale image labelled “02” using different algorithms with m=20,480 measurements. (**a**) is the original image “02”, (**b**–**g**) are the images retrieved by the DGI, GBGI, GHGI, GDGI, GLGI and GTGI, respectively.

Figure 3a shows a 64×64 grayscale image labelled “02”, whose gray value range is [0, 1]. With the same number of measurements, the above algorithms are executed again. Here, we set α=0.1. The results are presented in Figure 3b–g. The SNR and calculation time of the DGI is 3.86 and 2.59 s, respectively. In the recovered image of the DGI, the contour of the object is recognizable, but the background noise is messy and the detailed information is missing. As we can see, the GDGI acquires the best performance, with an SNR value of up to 32.70. The image retrieved by the GDGI contains only a small amount of noise and shows a great ability to resolve the details. In addition, the SNRs of the GBGI and GLGI are more than three times that of the DGI, while the GHGI’s performance was the worst (as it hardly acquires any useful object information) among these GGI variants due to the aforementioned HGI restrictions. The calculation time of the above methods is very close, with all less than 4 s, which further indicates that the GDGI can obtain high-quality reconstructed images, with the noise being well-suppressed at the expense of a negligible increase in computing time.

**Figure 4:**
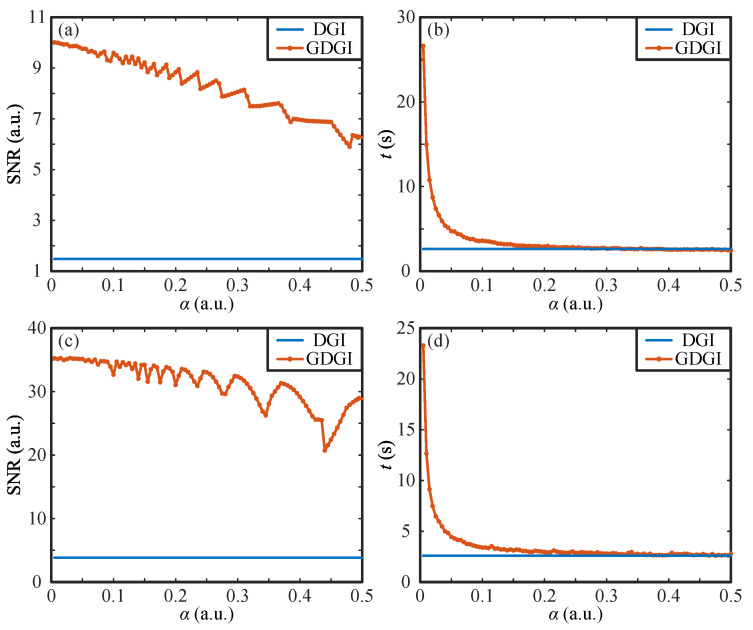
SNR and *t* curves as functions of the step size α. (**a**,**b**) and (**c**,**d**) are the curves for objects “01” and “02” by using the DGI and GDGI, respectively.

As mentioned above, the gradient descent results are greatly influenced by the step size α. Given that the GDGI has the best performance among the above variant algorithms, without loss of generality, we will mainly focus on the GDGI algorithm and discuss the relationship between the SNR and α value in depth. Take the binary image “01” and grayscale image “02” ranging from 0 to 1, as shown in Figure 2a and Figure 3a for examples, we drew the SNR curves of the GDGI as functions of the step size α as well as the calculation time *t* for the original images “01” and “02”, respectively, as shown in Figure 4a–d. The step size α was set from 0.005 to 0.5 with a 0.005 stepping increase, and, for each step size, both the SNR and calculation time were recorded.

As can be seen from Figure 4a,b, with the increase in the step size α, the SNRs of the reconstructed images of “01” present an oscillating downward trend, and the calculation time *t* decreases sharply and tends to be stable when α≥0.2. As we know, when the step size α is small, the gradient descend stepping distance of the each iteration is too small, so the convergence speed of iterations is slow and the calculation time is long. However, in this case, the algorithm is unlikely to miss the optimal solution in its iterative process, leading to a relatively high SNR with a high probability. When the step size α becomes large, the iterative convergence speed increases rapidly, but the iteration precision will drop, and there is a high probability that the optimal solution will be missed, causing a relatively low SNR. Furthermore, for some large α values, the current step of the iterations will cross the position of the optimal solution, which will lead to fluctuations, as shown in Figure 4a,c. Taking the DGI results of the binary image “01” (i.e., SNR = 1.48 and *t* = 2.62 s, see Figure 2b as a reference (which is drawn as straight lines in Figure 4a,b), it can be seen from the curves that no matter how the value of α changes in the range from 0 to 0.5, the SNRs of the GDGI are always higher than those of the DGI, and when α is greater than 0.1, the calculation time of GDGI is almost the same as that of the DGI. The above results demonstrate that our proposed method has a more efficient imaging performance than the DGI in the binary case.

For the grayscale image “02”, from Figure 4c,d we can see that the SNRs of reconstructed images present an overall downward trend and the calculation time *t* also drops sharply with the increase in step size α. When 0<α≤0.1, the SNRs of the GDGI are almost constant. When α further increases, the SNR gradually changes from a small-amplitude fluctuation to a large-amplitude fluctuation, as shown in Figure 4c. This is because the increase in the step size α causes an increase in the gradient descent stepping distance (when the iteration is close to the optimal solution but the preset termination conditions are not met, the jump will continue; the larger the step size, the more likely it is to jump out of the region where the optimal solution is located and enter a new reciprocating iteration, which may cause reciprocating oscillations in SNR values). Similarly, we took the DGI results of the grayscale image “02” (i.e., SNR = 3.86 and *t* = 2.59 s, see Figure 3b) as a reference (which is also drawn as straight lines in Figure 4c,d). We can see from Figure 4c,d that the SNRs of the GDGI never fall below those of the DGI, and when α is greater than 0.1, the calculation times of the GDGI and DGI are nearly the same. This proves that our scheme is also suitable for complex grayscale objects. According to the above results, to form a trade-off between the imaging quality and running time, the appropriate value range of the step size α should range from 0.05 to 0.15. For simplicity, in the following, we set α=0.1. In addition, since the result of F(O) is always positive, the iterative updating direction is usually opposite to the gradient direction, but we believe that α, in some special cases, can also take a negative value; for example, when the step size is too large, the algorithm misses the optimal solution and α should be changed to a negative value. This appears complicated and needs further study in the future, so it will not be discussed in this article.

**Figure 5:**
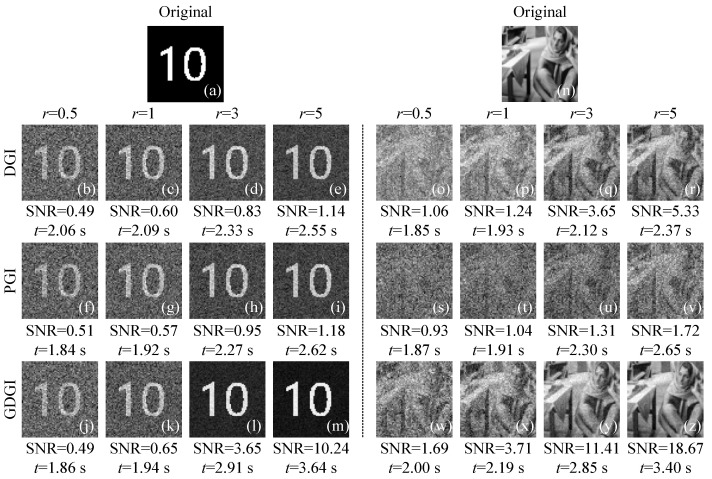
Simulation results of another two images “03” and “04” (all of 64×64 pixels) using the DGI, pseudo-inverse GI (PGI) and GDGI algorithms with r=0.5,1,3,5 sampling rates. (**a**,**n**) are the original images, (**b**–**e**) and (**o**–**r**), (**f**–**i**) and (**s**–**v**), (**j**–**m**) and (**w**–**z**) are the images recovered by the DGI, PGI and GDGI, respectively.

Next, to verify the universality and effectiveness of our algorithm, we tested another binary image and a grayscale image whose gray value range is [0, 1], marked as “03” and “04”, respectively. Figure 5 shows the original images and the corresponding imaging results of the DGI, PGI and GDGI with different sampling rates *r* (defined as the ratio of the number of measurements to the total number of image pixels). As can be seen from Figure 5b–m,o–z, when *r* = 0.5, the image quality of the PGI is slightly better than those of the DGI and GDGI for the image “03”, while the image quality of the GDGI is 0.59 and 0.82 times higher than those of the DGI and PGI for the image “04”. When *r* = 1, the image quality of the GDGI is 0.08 and 0.14 times higher than those of the DGI and PGI for the image “03”, and 1.99 and 2.57 times higher than those of the DGI and PGI for the image “04”. When *r* = 3 and 5, the imaging performance of the GDGI is much better than those of the DGI and PGI. Therefore, it can be inferred that, with the increase in sampling rate *r*, the improvement in image quality will be more significant. In addition, since our algorithm does not require preprocessing or optimizing of the measurement data, the use of a gradient-descent-like method can also fully improve the utilization of data, thereby greatly saving additional computational overhead. Here, the average running time of the GDGI is only 1.19 and 1.18 times those of the DGI or PGI, respectively, which means that a significant improvement in imaging quality can be achieved without much running time increment, i.e., our scheme has a relatively high return-to-investment ratio. Besides, through this simulation, it also demonstrates that our algorithm works well for different types of images.

**Figure 6:**
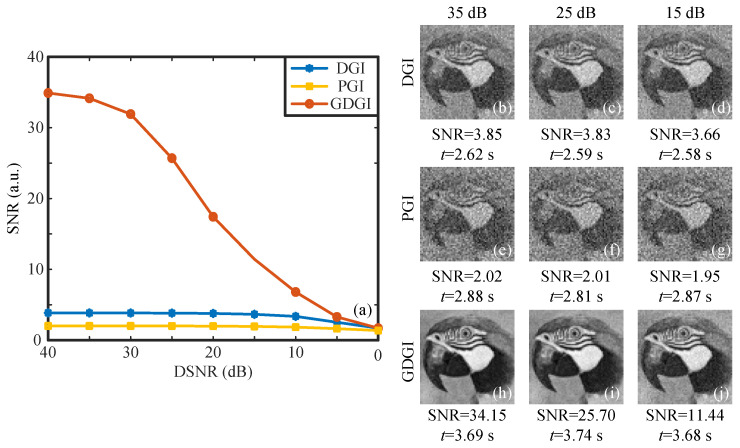
Simulation results for the grayscale image labelled “02” using the DGI, PGI and GDGI algorithms under different detection signal-to-noise ratios (DSNRs). (**a**) gives the changes in the SNRs of the DGI, PGI and GDGI with the DSNRs, (**b**–**d**), (**e**–**g**) and (**h**–**j**) are the images retrieved by the DGI, PGI and GDGI, with the DSNR being 35 dB, 25 dB, 15 dB.

In the following, we performed additional numerical simulations to test the robustness of the GDGI against noise. Taking the image “02” as an example, we further added white Gaussian noise to the bucket value $S_{B}$ to simulate the noisy measurements. Then, we calculated the SNRs of reconstructed images under different detection signal-to-noise ratios (DSNRs), i.e., the power ratio of the signal to the measurement noise, which can be expressed as $\mathrm{DSNR}=10\log_{10}\left(Var\left(S_{B}\right)/Var\left(S_{noise}\right)\right)$ [29], where $Var(S_{B})$ and $Var(S_{noise})$ denote the variances of the bucket values and measurement noise, respectively. As shown in Figure 6a, when the Gaussian random noise with different variances is added to the bucket signal, the SNRs of images recovered by the DGI, PGI and GDGI decrease with the decrease in the DSNR. It can be clearly seen from the curves that when the DSNR is greater than 5 dB, the imaging performance of the GDGI is significantly better than those of the DGI and PGI; when the DSNR is less than 5 dB, the SNRs of these three methods become closer, but the SNRs of the GDGI are still slightly higher than those of the DGI and PGI. This is because the statistical averaging of the intensity correlation is accompanied by reconstruction noise. The lower the DSNR, the harsher the measurement condition and the more difficult it is to distinguish the useful signal from the noise, which leads to a larger reconstruction noise and makes it more difficult to recognize the object part in the image. In ultra-low DSNR conditions, all algorithms including convex optimization will tend to fail. As shown in Figure 6b–j, we made some comparisons among the DGI, PGI and GDGI, with the DSNR changing from 35 dB to 15 dB. When DSNR = 35 dB, 25 dB and 15 dB, the SNRs of the GDGI are 8.87, 6.71 and 3.13 times those of the DGI, and are 16.91, 12.79, 5.87 times those of the PGI, showing that our algorithm has a good anti-noise ability compared with the traditional GI algorithm under noisy measurements.

## 4. Experimental Demonstration and Discussions

### 4.1. Experimental Setup

**Figure 7:**
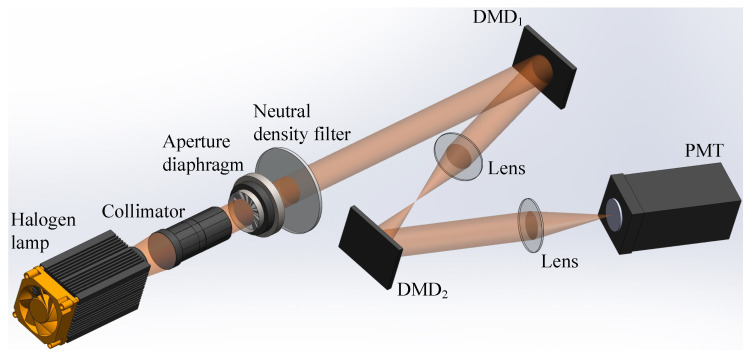
Experimental setup of computational GI. DMD: digital micromirror device, PMT: photomultiplier tube.

To further verify the effectiveness of the proposed architecture, we performed an optical experiment based on a classic single-arm computational GI setup, as shown in Figure 7, where a digital micromirror device (DMD) was used as an SLM and a counter-type Hamamatsu H10682-210 photomultiplier tube (PMT) was used as a bucket detector. The thermal light emitted from a halogen lamp passed through a collimator, an aperture diaphragm and a neutral density filter. Then the light beam illuminated the first DMD (indicated as ${\mathrm{DMD}}_{1}$) and was modulated by the preset binary patterns. After that, the modulated (structured) light was projected onto the second DMD (indicated as ${\mathrm{DMD}}_{2}$) through the imaging lens, where the digital object was displayed onto ${\mathrm{DMD}}_{2}$. The light reflected by ${\mathrm{DMD}}_{2}$ was converged by the lens onto the bucket detector. The detector recorded the sequence of the total intensity in the form of photon counts, denoted as $S_{B}=\left\{S_{B}^{1},S_{B}^{2},\cdots ,S_{B}^{j},\cdots ,S_{B}^{m}\right\}$ (corresponding to different modulated binary patterns). In the following experiments, DGI and GDGI are the two main algorithms to be considered and compared.

### 4.2. Experimental Results of a Binary Object

**Figure 8:**
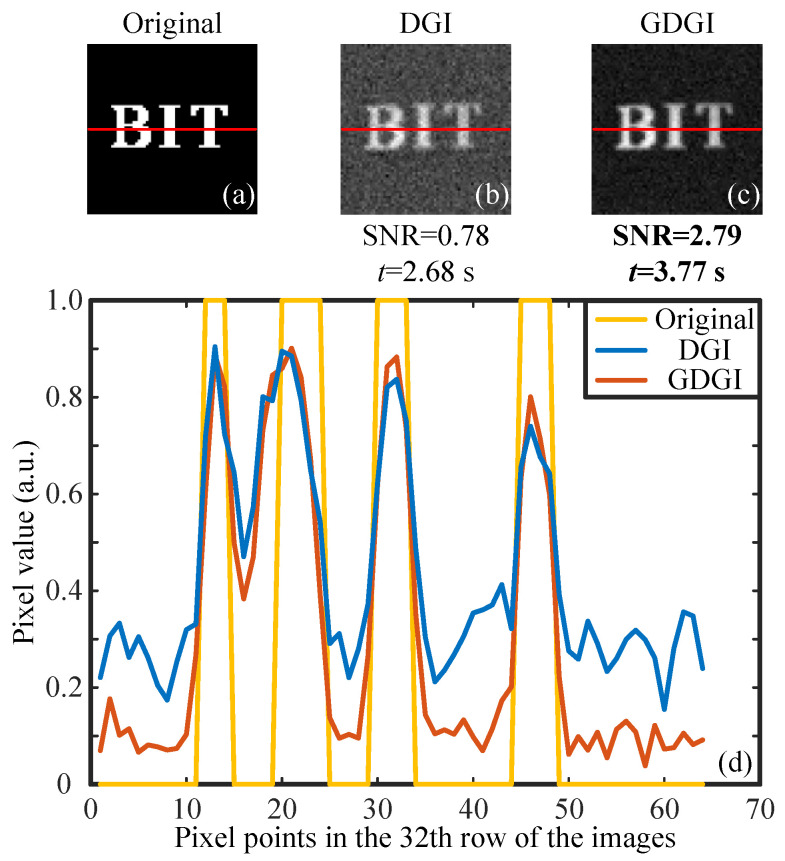
Experimental results of a binary object. (**a**) is the original image encoded onto ${\mathrm{DMD}}_{2}$, (**b**,**c**) are the experimental results recovered by the DGI and GDGI, and (**d**) gives cross-section plots of the grayscale images in the 32th row.

For the imaging experiments of a binary object, we encoded a 64×64 binary image “BIT” onto ${\mathrm{DMD}}_{2}$ as the original object image (see Figure 8a), then presented the reconstructed images with r=5 (as an example) in Figure 8b,c. The SNRs of the DGI and GDGI are 0.78 and 2.79, respectively. It is obvious that the latter is 3.58 times the former, but the calculation time of the latter is only 3.77 s, consistent with the aforementioned simulation results. Furthermore, the visibility of the GDGI is much better than that of the DGI. To make a more intuitive comparison of the reconstructed images, we plotted the curves of the grayscale values in the 32th row (consisting of 64 pixels) of the original image and restored images (marked by the red lines in the Figure 8a–c. It can be clearly seen from Figure 8d that our GDGI can significantly reduce the background noise and improve the contrast compared with traditional DGI, thus dramatically improving the visibility. Without loss of generality, this also indicates that our GGI scheme can efficiently improve the SNRs of GI reconstructions.

**Figure 9:**
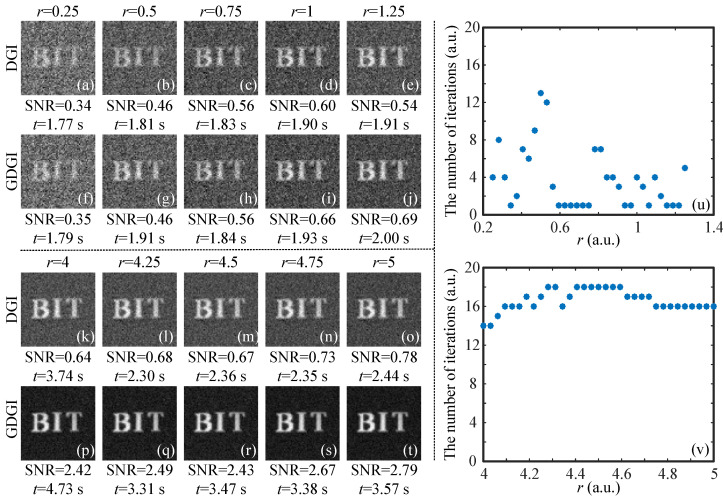
Comparison results of the binary object “BIT”. (**a**–**t**) are the reconstructed images of the DGI and GDGI using different sampling rates; (**u**,**v**) give the change in the number of iterations with the sampling rate, all using the GDGI.

Next, the images of the DGI and GDGI were reconstruced using different sampling rates (see the left half of Figure 9). Above the dotted line on the left half of the graph, we increased the sampling rate *r* from 0.25 to 1.25, step by 0.25, and below the dotted line, we made *r* change from 4 to 5, also step by 0.25. It is easy to see from Figure 9a–t that when *r* is smaller than 1, the GGI benefits are small, and when *r* is larger than 1, the gains become increasingly apparent. The reason for this is that when the measurement data are relatively limited, it is difficult for a finite number of iterations to maximize the performance gains; when the measurement data are rich, these iterations can effectively take advantage of the correlation within the data to suppress noise and improve image quality. To further demonstrate the performance of the proposed method, the relationship between the number of iterations it takes for the algorithm to terminate and the sampling rate is given on the right half of Figure 9. As shown in Figure 9u, when *r* is between 0.25 and 1.25, most of the iteration numbers required for the algorithm to converge are lower than 8. In this case, the GGI fails to obtain effective performance gains. However, when *r* is between 4∼5, the number of iterations required for convergence is about 16, as shown in Figure 9v. In this situation, the iterations gradually approach the position of the optimal solution, and thus the image quality is significantly improved. Therefore, it can be concluded that, in our algorithm, the image quality can be effectively improved with a limited number of iterations without any preprocessing or optimization.

**Figure 10:**
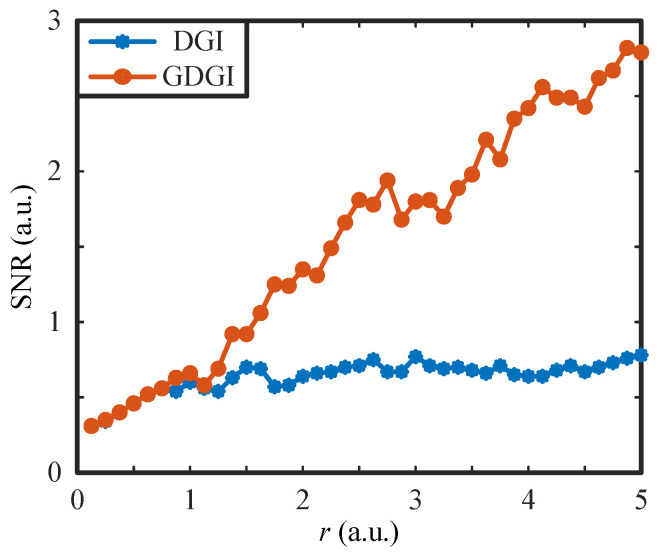
SNR values of the images reconstructed by the DGI and GDGI for the binary object “BIT” with different sampling rates *r*.

To more clearly see the change in the SNR values, we plotted the SNR curve as a function of the sampling rate *r*, as shown in Figure 10 (here, we took the sampled data corresponding to the original image “BIT” for investigation). One can see clearly that, with the increase in the sampling rate *r*, the SNRs of the GDGI increase more sharply than those of the DGI. Therefore, our nonlinear algorithm has a better performance than the linear algorithm.

### 4.3. Experimental Results of a Grayscale Object

**Figure 11:**
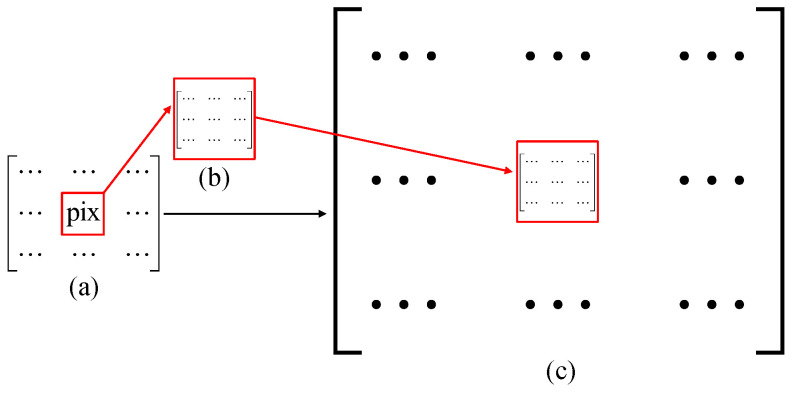
Schematic diagram of encoding a grayscale image onto a DMD. (**a**) shows the pixel size for reconstruction, (**b**) gives an example of an pixel-unit “pix”, and (**c**) is the actual pixel size displayed on the DMD.

In the experiment, we use the image displayed on ${\mathrm{DMD}}_{2}$ as the object. The DMD is composed of millions of micromirrors (pixels), each of which orientates $\pm 12^{\circ }$ with respect to the normal direction of the working plane, corresponding to two states (1 or 0). Thus, the matrix encoded onto the DMD should be binary, consisting of 1 and 0. To our knowledge, to display grayscale objects, the pulse-width modulation (PWM) strategy, which exchanges time-pulse width for grayscale level, is generally used. Since the DMD should sequentially switch multiple binary patterns for each grayscale image’s display, the higher the grayscale level, the more time the PWM takes. Here, we chose to apply another method called Floyd–Steinberg error diffusion dithering [41] to convert the grayscale image to binary image. As shown in Figure 11, to reconstruct a grayscale image of 64×64, we need to apply upsampling to this image and set its each pixel as a pixel-unit “pix” consisting of 12×12 pixels (i.e., a grayscale pixel-unit occupies 144 mircormirrors in total with a gray value ranging from 0 to 144). The pixels are randomly lit up (set to 1) in a pixel-unit; the number of bright (1) pixels indicates the gray value of this pixel-unit. Here, for a 64×64 grayscale image’s display, a matrix of 768×768 actual pixels will be loaded onto ${\mathrm{DMD}}_{2}$.

**Figure 12:**
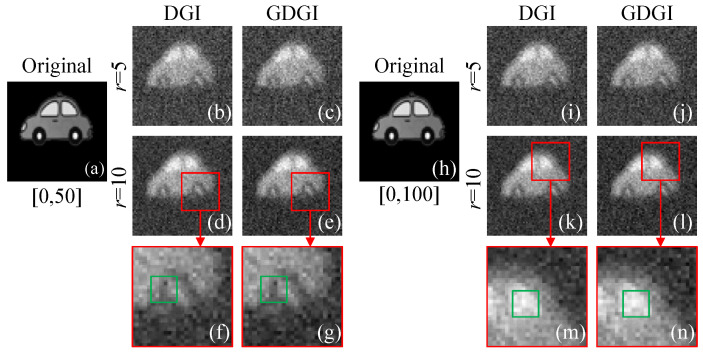
Experimental results of a grayscale object. (**a**,**h**) are the original object images “car” that are encoded onto ${\mathrm{DMD}}_{2}$, with grayscale ranges of [0, 50] and [0, 100], respectively; (**b**–**e**) and (**i**–**l**) are the experimental results recovered by the DGI and GDGI, respectively; (**f**–**g**) and (**m**–**n**) are the enlarged detailed images of (**d**–**e**) and (**k**–**l**), respectively.

In this experiment, we encoded two grayscale images onto ${\mathrm{DMD}}_{2}$ “car” (see Figure 12a,h) with a grayscale range of [0, 50] and [0, 100], respectively. Their experimental results using the DGI and GDGI under the sampling rate *r* = 5 and 10 were given in Figure 12b–e,i–l. It is obvious that the reconstruction results of *r* = 10 are better than those of *r* = 5, and the reconstructed details for a larger grayscale range are richer. To compare the details of the reconstructed images more intuitively, we enlarged the image regions within the red rectangles of Figure 12d–e,k–l, and drew green rectangles in the corresponding areas for more detailed observation, as shown in Figure 12f–g,m–n. Through subtle comparisons, we found that the GDGI will achieve a higher visibility than the DGI, but the improvement effects are not obvious. In this experiment, the measured bucket (single-pixel) signal is accompanied by a lot of noise, the DSNR is very low, and the GDGI algorithm terminates after only 1∼6 iterations; thus, the gain of the GDGI is small. The acquisition of high-quality images under low DSNR conditions is a tough problem for GI, and this will be the focus of our future research.

## 5. Conclusions

In conclusion, we present an effective GI reconstruction method based on gradient-descent-like strategy, which can use an intensity correlation function in its iterative formula. Both numerical simulation and optical experiments have been performed to demonstrate the performance of this proposed method. By setting the appropriate step size, this method can easily acquire an optimal solution through only a few iterations with low computational complexity. Moreover, it can also deal with both binary and grayscale complex objects in a slightly noisy environment, and suppress reconstruction noise to a certain extent. We find that the GDGI, as a variant of our GGI method, outperforms the other variants and can achieve a much better performance than that of the DGI, especially for binary complex objects, at the expense of a negligible increase in running time caused by iterations. Therefore, we believe that this new iterative calculation scheme may pave the way for the intensity correlation from the linear statistical average towards the nonlinear one, which will provide more possibilities for GI reconstructions.
